# Isolated Pancreatic Neck Injury Due to Motorbike Accident

**DOI:** 10.7759/cureus.70340

**Published:** 2024-09-27

**Authors:** Fidan Huseynova, Mirjavad Abdullazade, Zohre Abdullazada, Emin Bayramov, Altay Aliyev, Elgun Samadov

**Affiliations:** 1 Department of General Surgery, Ondokuz Mayıs University, Samsun, TUR; 2 Department of General Surgery, Koç University, Istanbul, TUR; 3 Department of Surgery, Liv Bona Dea Hospital, Baku, AZE; 4 Department of Oncology, Liv Bona Dea Hospital, Baku, AZE

**Keywords:** blunt abdominal trauma, distal pancreatectomy, isolated pancreatic trauma, management of pancreatic injuries, pancreas, pancreatic injuries, splenectomy

## Abstract

Isolated pancreatic injuries that result from blunt abdominal trauma are a rare occurrence due to the retroperitoneal location of the pancreas that shields the pancreas from trauma. The nonspecific clinical presentations of patients can delay the diagnosis and complicate the decision in patient care. It is crucial that a high degree of suspicion is maintained for pancreatic injuries in cases like this one. For optimal patient care, a multidisciplinary approach should be taken, and advanced imaging techniques, such as contrast-enhanced computed tomography (CT), should be used.

The patient is a 26-year-old male who was involved in a motorbike accident. The patient was initially treated in a nearby small-town hospital. The patient was transferred to our medical facility four days after the accident took place due to clinical deterioration. He complained of severe generalized abdominal pain that was especially prominent in the left upper quadrant. Initial contrast-enhanced CT scans indicated a grade III full-thickness transection of the pancreatic body and tail. Despite the initial conservative treatment, the patient's hemodynamics were unstable, and the decision for surgical intervention was made. A distal pancreatectomy and splenectomy were performed, and the patient's recovery was uneventful.

This case points out the challenges that a physician might face when diagnosing isolated pancreatic injuries that result from blunt abdominal trauma. For an accurate diagnosis, advanced imaging techniques, such as contrast-enhanced CT, should be used. A multidisciplinary approach should be taken to provide the patient with optimal medical care. Finally, this study emphasizes the need for standardized transfer protocols in resource-limited settings.

## Introduction

Pancreatic injuries that result from abdominal trauma are a rare occurrence that accounts for a very small portion of cases of abdominal trauma. According to a study, only 3.1% of patients who had documented abdominal trauma had a pancreatic injury [1]. One of the contributing factors to this is the retroperitoneal location of the pancreas, which shields the pancreas from abdominal trauma. The symptoms of these injuries are nonspecific, and patients can present with nonspecific clinical manifestations that may delay the diagnosis and patient care [2]. Advanced imaging techniques, especially contrast-enhanced computed tomography (CT) scans, are required for timely and accurate diagnosis [3]. Early diagnosis of these injuries are crucial for guiding optimal care for the patient, which may range from conservative management to surgical intervention depending on the extent of the injury and the hemodynamic state of the patient. Although pancreatic injuries caused by abdominal trauma are a rare occurrence, it poses an average mortality rate of 10%-30%, which is a major concern [4]. This case study will discuss a patient who sustained a pancreatic injury due to abdominal trauma. Through this case, we aim to clarify the various challenges encountered both in the diagnosis and management of this injury.

## Case presentation

A 26-year-old male patient who was involved in a motorbike accident was transferred to our hospital four days after the accident took place. The patient was initially treated at a small-town hospital, his symptoms worsened, and he had to be transferred to our facility. Upon presentation, the patient reported severe generalized abdominal pain that was most prominent in his left upper quadrant. Additionally, the patient also reported nausea, fatigue, and generalized weakness. His vital signs were as follows: blood pressure (BP) of 120/70 mmHg, heart rate (HR) of 92 beats per minute (bpm), respiratory rate (RR) of 14/minute, temperature of 37.1°C, and oxygen saturation (SpO2) of 97% on ambient air.

On physical examination, the patient was cooperative and oriented to time and place. The patient's Glasgow Coma Scale score was 15/15. The patient's pupils were equal, round, and reactive to light. His sclerae were non-icteric and non-injected. His general appearance was notable for pallor, with bruising on the face and especially on the right eye. The oropharynx was clear, and mucous membranes were moist. During auscultation of the lungs, decreased breath sounds were heard at the left lung base. However, the patient's breathing was non-labored, and his oxygen saturation was normal. During palpation of the abdomen, a generalized severe tenderness was noted that was particularly prominent in the left upper quadrant; furthermore, the abdomen was mildly distended. During examination of the skin, there were no spider nevi, no palmar erythema, no rashes, and no lesions, and the skin turgor was normal. Despite his symptoms, the patient was hemodynamically stable. For further investigations, consultations were requested from infectious diseases and general surgery. For close monitoring and further management, the patient was admitted to the intensive care unit (ICU).

Upon admission, the patient had undergone the following diagnostic tests: complete blood count (CBC); liver function tests (LFTs); procalcitonin (PCT); C-reactive protein (CRP); viral serology for hepatitis B, hepatitis C, HIV, and syphilis; amylase; lipase; creatinine kinase (CK); bilirubin; albumin; creatinine; estimated glomerular filtration rate (eGFR); blood urea nitrogen (BUN); urinalysis; urine culture; blood cultures (for both aerobes and anaerobes); and stool cultures. Important findings are indicated in Table 1 and Table 2. All other tests not listed in Table 1 and Table 2 are normal.

**Table 1:** Laboratory test results for the case WBC: white blood cells, RBC: red blood cells, HGB: hemoglobin, HCT: hematocrit, PCT: procalcitonin, PLT: platelets, CK: creatinine kinase, CRP: C-reactive protein

Laboratory finding	Results (at presentation)	Results (postoperative day 1)	Reference range
WBC (K/uL)	13.20	19.31	4.5-11
RBC (M/uL)	3.13	3.38	4.35-5.65
HGB (g/dL)	9.5	10	14-18
HCT (%)	28.1	30.1	40-54
Neutrophil count (K/uL)	11.48	14.7	2-7
Lymphocyte count (K/uL)	0.75	1.74	1-4.8
PCT (ng/mL)	0.34	-	<0.1
PLT (K/uL)	179	512	3.5-5.5
CK (U/L)	1,104	-	22-198
Bilirubin, total (mg/dL)	1.4	1	0.1-1.2
Bilirubin, direct (mg/dL)	0.4	0.2	<0.3
CRP (mg/L)	318.54	211.52	<0.3

**Table 2:** Urinalysis results for the case HPF: high-powered field

Components	Patient's results	Reference ranges
Color	Yellow	Yellow/amber/red/blue/colorless/straw
Clarity	Clear	Clear/slightly cloudy/cloudy/turbid
Specific gravity	1.050	1.005-1.030
pH	7	5-9
Nitrite	Negative	Negative
Protein	+2 Positive	Negative
Glucose	Negative	Negative
Ketone	+1 Positive	Negative
Red blood cells (HPF)	69	0-3
White blood cells (HPF)	248	0-2

**Figure 1:**
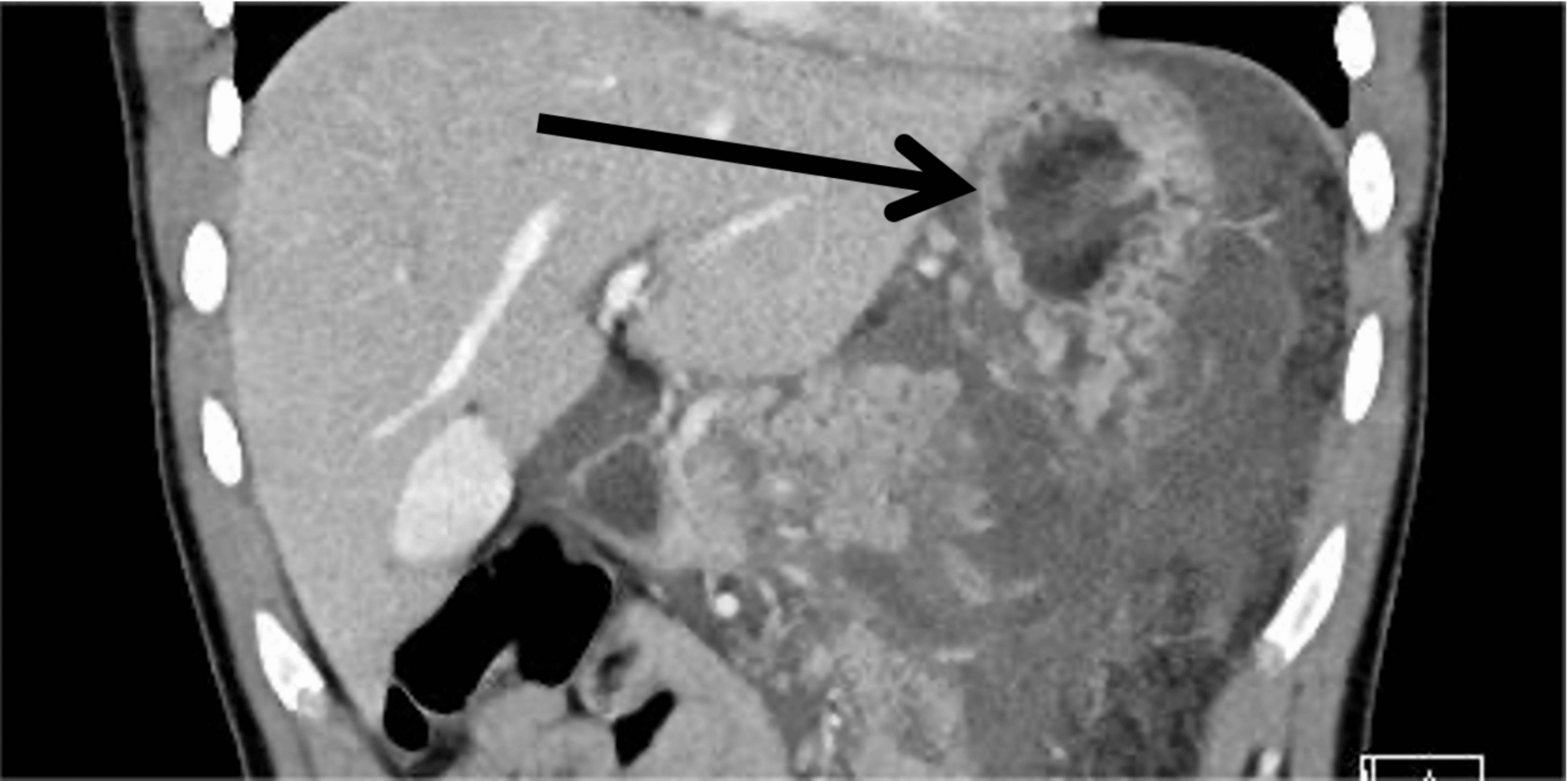
Contrast-enhanced CT scan (coronal view) The black arrow indicates the transection of the distal pancreas. CT: computed tomography

**Figure 2:**
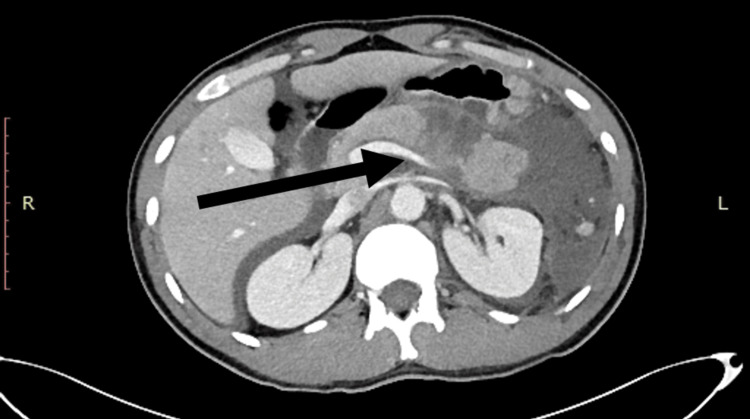
Contrast-enhanced CT scan (portal view) The black arrow indicates a full transection of the distal part of the pancreas. CT: computed tomography

The patient underwent cranial, thoracic, and abdominal contrast-enhanced CT scans. The results showed that the pancreas had a full-thickness injury transection at the distal part, body, and tail of the pancreas. The rupture was evaluated to be a grade III injury in the portal and coronal sections of the CT image (Figure 1 and Figure 2). The abdomen, pelvis, gastrosplenic space, subhepatic area, and intestinal loops were surrounded by free fluid. During the evaluation, the patient's vital signs were normal, so we decided to proceed with conservative follow-up. However, after two days of conservative treatment, the patient had decreased hemoglobin (HGB) at 8 g/dL and was hemodynamically unstable, and the decision for an exploratory laparotomy was made. The patient was placed under general anesthesia, and a midline laparotomy incision was made.

**Figure 3:**
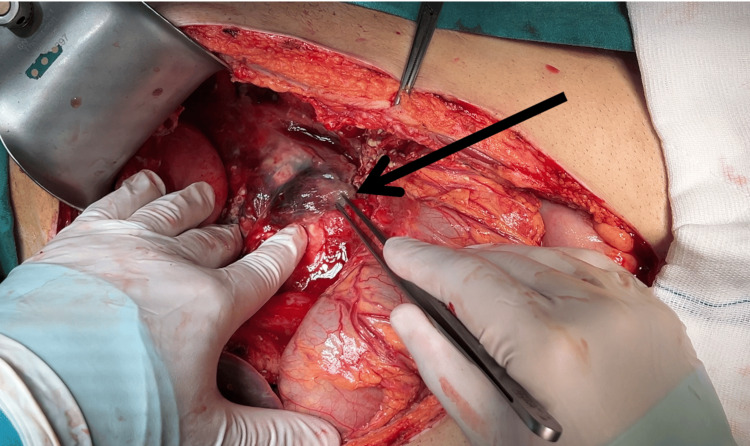
Subcapsular hematoma is seen in the transected distal pancreas (black arrow)

**Figure 4:**
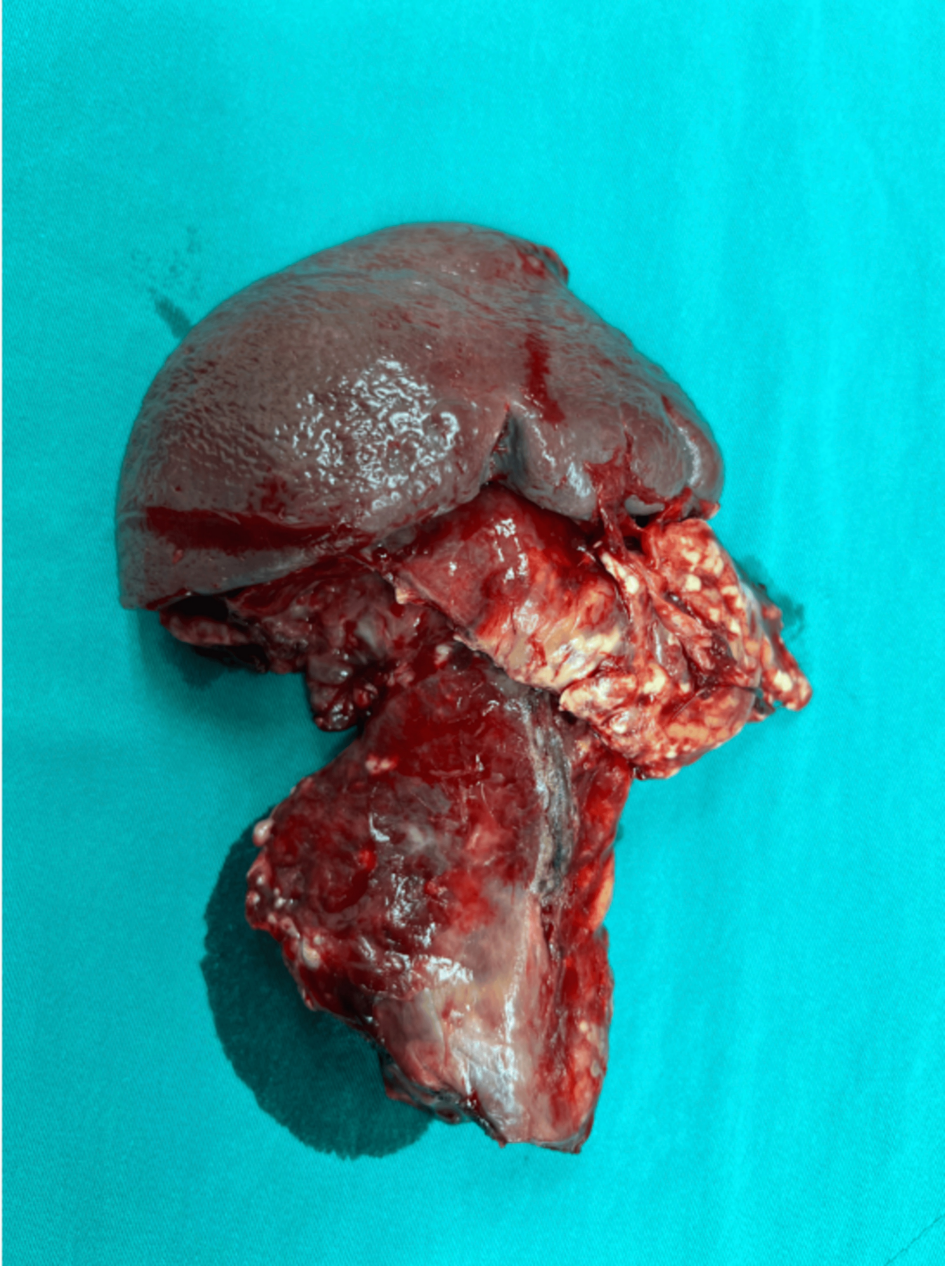
Excised spleen and pancreas

During exploration of the peritoneal cavity, 1,200 mL of hemorrhagic free fluid was aspirated. A subcapsular hematoma was detected in the transected distal pancreas (Figure 3) and was excised. Traumatic rupture and separation of the pancreatic body was noted. The separated distal pancreatic body and the spleen was excised (Figure 4). Splenic artery and vein were ligated and excised, leaving the pancreas stump intact. The pancreas stump was sutured twice with 3-0 prolene sutures. After a thorough exploration, the peritoneal cavity was irrigated with antiseptic solution, and Jackson-Pratt (JP) drains were placed in the pelvis, subhepatic area, and subgastric bed. All surgically removed materials were transferred to the pathology laboratory for additional examination following the excision of the spleen, surrounding lymph nodes, and the distal pancreas. According to the pathology reports, the tissues revealed an inflammatory response, which was expected given the patient's injury. The patient's recovery was uneventful, and he was discharged three days after the operation. He was regularly followed up in the clinic, vaccinations were done due to splenectomy, and regular screening was done to rule out possible complications.

## Discussion

Pancreatic injuries following blunt abdominal trauma are uncommon, accounting for less than 2% of abdominal injuries. They present a significant challenge in diagnosis and management to clinicians due to the pancreas' deep retroperitoneal location and the typically nonspecific nature of early symptoms [5]. The case study presented here underlines the complex situation where the patient presents four days after the accident with nonspecific and delayed symptoms. The initial presentation of generalized abdominal pain accompanied by nausea, fatigue, and stable signs leads the management to a more conservative approach; however, this case highlights the importance of monitoring and readiness to escalate treatment when necessary [6,7]. Adding to the rarity of isolated pancreatic injuries, pancreatic injuries are often accompanied with injuries to other abdominal organs, making it even more challenging to diagnose and manage these patients. This case illustrates the importance of considering isolated pancreatic injury in the differential diagnosis of patients with blunt abdominal trauma.

Usage of advanced imaging techniques greatly aids the diagnosis and management, especially contrast-enhanced CT scans, which are considered the mainstay of primary detection of pancreatic injuries [8]. Contrast-enhanced CT scan revealed a full-thickness transection of the distal part, body, and tail of the pancreas, which is classified as a grade III injury according to the American Association for the Surgery of Trauma (AAST) classification system [9]. The patient was managed in correlation to the current guidelines, which state that patients with stable hemodynamics and without any alarming symptoms should be treated conservatively [10,11]. However, conservative management of the patient's condition could not be maintained due to the patient's clinical deterioration, which prompted a surgical intervention. The decision to move on to surgery was challenging due to the increased morbidity and mortality that is associated with surgical intervention in pancreatic injuries; nevertheless, the patient's clinical condition was deteriorating rapidly, and it was the best viable option for us to consider [12]. It is crucial that the surgery is undertaken by experienced hepatobiliary surgeons in centers capable of handling such patients [13].

This case study primarily addresses the important issue of managing severe abdominal trauma in areas devoid of a standard medical infrastructure. Given the lack of advanced medical tools and standardized medical practices, the diagnosis and management for pancreatic injuries become more challenging. As this study shows, the absence of alarming symptoms does not exclude major underlying injury. This instance emphasizes the need for extensive testing in individuals with blunt abdominal trauma even if these patients are displaying nonspecific symptoms and a normal physical examination. Despite extensive research, we discovered that there is a lack of studies on the diagnosis and treatment of pancreatic injury in settings with limited resources. This emphasizes the need for developing standardized processes to direct the management and transfer of these patients to more advanced institutions. These systems ought to be carefully built to strike a compromise between best utilization of resources and improvement of patient outcomes. More study on this matter is absolutely vital to ensure every patient may get the finest possible quality of treatment.

## Conclusions

This case study highlights the importance of diagnosing and managing isolated pancreatic injuries that occur as a result of blunt abdominal trauma. The severity of the injury was masked due to the nonspecific presentation and stable condition of the patient. However, the extent of the injury was diagnosed using contrast-enhanced CT scans. An initial conservative management was planned; however, due to the patient's clinical deterioration, the decision for surgical intervention was made. The patient's recovery was uneventful, and the patient was followed up in regular clinic visits for the next three months. Apart from the complexity of the injury and the challenges faced in the management of this patient, this case emphasizes the need for implementing standardized protocols for the management and, if possible, transfer to an advanced medical facility. After thorough research, we determined that there was a lack of research regarding the management of complex trauma cases like this one in resource-limited settings. With standardized protocols, we could ensure that all improved patient outcomes while also preserving resources.
